# "The Hospital" Nursing Mirror

**Published:** 1897-06-05

**Authors:** 


"The Hospital" Nursing Mirror.

Being the Nursing Section of "The Hospital."

[Contributions for this Section of "The Hospital" should be addressed to the Editor, The Hospital, 28 & 29, Southampton Street, Strand, London, W.C., and should have the word "Nursing" plainly written in left-hand top corner of the envelope.]

### News from the Nursing World.

### THE IRISH FUND FOR THE QUEEN'S NURSES.

A very largely-attended meeting in aid of the Fund for providing Queen's Nurses for the Irish poor was held in Dublin last week in the Leinster Lecture Hall, at which Mr. John E. Barry, president of the Chamber of Commerce, presided. The following resolution was proposed by the Earl of Meath in an excellent speech: "That the pressing need of trained nurses to visit the sick poor in their own homes makes it most expedient that the Diamond Jubilee Memorial Fund in Ireland should be sufficient, not only to permanently strengthen the system of Queen's Nurses in the Dublin District, but to aid localities throughout the country which have hitherto been unable to adopt it from want of such assistance." Miss Dunn, superintendent of the Irish branch of the Queen's Jubilee Institute for Nurses, seconded the motion, and gave an interesting explanation of the work of the district nurses, and the needs of the institute. The resolution was adopted with much applause, and others were carried, earnestly inviting contributions to the Fund, and also arranging for the presentation of a loyal address to the Queen.

### BARRY DISTRICT NURSING ASSOCIATION.

The Barry District Nursing Association reports fifty-seven new cases during April, 1,823 visits having been paid by the staff of four nurses in the course of the month. The Accident Hospital (five beds) also did good work. Ten accidents were admitted; others were sent to Cardiff, owing to want of accommodation. The accidents happen chiefly to the men, of whom there are some three or four thousand, working on the extensive new docks that are being built.

### MRS. GARRETT ANDERSON AT IPSWICH.

Mrs. Garrett Anderson, M.D., as president of the East Anglian branch of the British Medical Association, on Thursday, May 27th, delivered an address before the members of the association at Ipswich, the first time in the history of English medicine that a woman has occupied such a position. Mrs. Anderson took for her subject the progress of medicine in the Victorian Era, reviewing generally the tremendous changes which the past sixty years have seen, and concluding a very interesting speech by forecasting something of the lines on which medical science might be expected to develop itself in the future.

### A WOMEN'S MEMORIAL.

A memorial largely signed by influential women has been presented to Lord Salisbury, Lord George Hamilton, and other members of the Government, expressing an anxious hope that "effectual measures will be taken to check the spread of contagious diseases among our soldiers, especially in India." The petition ends with these words: "And with the deepest earnestness we call on the Government to do all that can be done to save innocent women and children in the present and future generations from the terrible results of vices for which they were not responsible." The first three signatories are T.H.R. Princess Christian the Duchess of Connaught, and the Duchess of Teck. Then follow the names of many distinguished women, including those of the matrons of many large London hospitals. Miss Florence Nightingale and Mrs. Humphrey Ward sign with the reservation that independent inquiries shall be set on foot at the several stations in India.

### THE NURSES' CO-OPERATION.

The Committee of the Nurses' Co-operation have lost no time in appointing a successor to Miss Philippa Hicks, and are to be much congratulated upon the choice they have made in Miss Amy Hughes. Miss Hughes, the details of whose nursing career will be found in another column, did specially good work during the years she filled the position of Superintendent at the Central Training Home of the Metropolitan Nursing Association in Bloomsbury Square, and her resignation and departure to Bolton were felt to be a real calamity by all concerned. The experience there gained will, however, doubtless prove valuable to Miss Hughes in her new capacity as the head of so successful an institution as the Nurses' Co-operation, and she will certainly receive a very warm welcome back to London from her many friends.

### NURSING NOTES "QUEEN'S NURSES' NUMBER."

The editors of *Nursing Notes* are to be congratulated upon the excellence of the June number, of which we have just received a copy. It is a "Special Queen's Nurses' Number," and so of course a good deal of space is devoted to the work of Queen Victoria's Jubilee Institute for Nurses, the place of honour being given to an article by the President of the Institute, the Rev. A. L. B. Peile. In the columns devoted to Workhouse nursing matters will be found a review of the history of the Workhouse Infirmary Nursing Association, a society which has done so much in its special field during the 18 years of its existence. It is a number full of valuable information.

### CAPS AND APRONS.

A delightful change has been effected in the uniform of the nursing staff at Guy's Hospital. On the great occasion of the Prince of Wales's visit to open the Medical School buildings last week, for the first time head nurses and probationers wore the new caps (whose advent has been the subject of much interesting speculation for some time past according to the *Guy's Hospital Gazette*), and aprons with bibs and straps. The linen collar is fastened with a heliotrope bow, toning with the lilac print dresses. The new caps are very neat and pretty, and not quite like any we have seen; they are, in fact, of Miss Nott-Bower's designing, and are made of lawn, in shape something of the Sister Dora, with plain band and full crown, gathered in by a string at the back. The "pro.s'" caps are stringless, while those of the head nurses fasten under the chin with a dainty little bow. The lady pupils, who have hitherto worn aprons without bibs, are to have these essential additions almost directly, and great, we are sure, must be their rejoicing thereat. Miss Nott-Bower introduced print dresses for the lady pupils and the sisters some time since; the days are not so long past when the former wore black cashmere dresses, and were required by regulation to carry some inches of trailing skirt behind them. Straps to the sisters' aprons, the bibs of which are now pinned in ancient fashion, and a reconstruction of the very particularly ugly headgear of the lady pupils, are all that is needed to make the uniform of the entire staff as neat and becoming as could be wished.

### THE JUBILEE AND NURSES' HOLIDAYS.

We are very glad to see that several institutions are making an effort to increase the annual leave of the nursing staff by way of celebrating this memorable year. The secretary of the Dewsbury and District General Infirmary writes to tell us that the House Committee of that institution have granted their nurses an extra week's holiday, and authorised the matron to arrange for additional leave at her discretion for the servants. One cannot but hope that the extra week thus given may in some instances, where leave is short as a general rule, become a permanent addition to the annual holidays.

### NURSES AND THE PLAGUE IN INDIA.

News from India shows that the plague is decreasing in a satisfactory manner, and at Bombay it has been found possible to close several of the hospitals. Of the nurses sent out from England, six are now at the Parel Hospital, six at the Arthur Road Hospital, and six at the Grant Road Hospital, while others are at Poona and elsewhere. The three hospitals mentioned will be kept open during the monsoon.

### HOSPITAL FOR EPILEPSY AND PARALYSIS.

A pleasant entertainment was got up for the patients at the Hospital for Epilepsy and Paralysis, Regent's Park, on May 26th, by Mr. Oscar Berry and a party of friends. A good programme was successfully carried through, including songs and recitations, and that very amusing duologue, "A Pair of Lunatics," to wind up with, given by Mr. Oscar Berry and Miss Edith Spiers.

### A NURSES' FRIEND.

Mr. Edward Gibbon, of Gateacre, who has recently died, has been for many years a good friend to the cause of nursing and hospital reform. For 42 years he was chairman of the Royal Infirmary, Liverpool, in which town he worked unceasingly for the advancement of trained nursing from the days of its first beginnings, when he came to London to see for himself what Miss Nightingale was doing for nurses and their work, and returned to Liverpool to do what he could to help forward a similar improvement. Mr. Gibbon was the first chairman of the Liverpool Training School and Home for Nurses.

### GWELO BOUND.

The three English nursing sisters, Miss Harding Miss H. Kenealy, and Miss Muriel, who sailed from Southampton on May 1st, in the R.M.S. *Dunvegan Castle, en route* for the Chartered Company's Hospital at Gwelo, arrived at Cape Town on the 18th ult. The voyage was accomplished in delightful weather, and passed off most pleasantly. Among the passengers were several nurses going out to Johannesburg for private work, and Captain Thorne, a son of Dr. Bezly Thorne, was also returning to South Africa. The nursing trio had hoped to see some of the beauties of Cape Town, but were able to devote only 24 hours to this as the trains to Mafeking run only once a week. So they continued their journey to Mashonaland on the 21st, and will have the novel experience of several days' travel in a bullock wagon before reaching their destination at Gwelo, where their arrival is eagerly waited for.

### ZENANA MEDICAL MISSION.

An interesting little *brochure*, "Pictures of Our Work," has just been issued by the Zenana Bible and Medical Mission, which gives an English reader a very good idea of the work of the missioners in India. Amongst the illustrations is one of a group at the Lucknow Hospital, in the centre of which are the two resident English medical women, Miss Haskew and Miss Cornall, with their colleagues, Miss Townsend and Nurse Sheldon. The society's workers have had a heavy time this year, what with famine and plague and the consequent distress, and their resources must have been taxed to the uttermost.

### SHORT ITEMS.

Nurses will be glad to know that the Trained Nurses' Club, 12, Buckingham Street, Strand, W.C., will be open on Jubilee Day at 9 a.m., and that not only members of the club but other nurses and members' friends will be allowed the use of the rooms for leaving cloaks and bags, and for resting between the procession and the illumination, at the small charge of 1s. Tea can be obtained.—H.R.H. Princess Henry of Battenberg has consented to become patroness of the Colonial Nursing Association.—A meeting for nurses was held under the auspices of the Women's Total Abstinence Union, by invitation of Lady Battersea, on the 26th inst., at Surrey House. Lady Battersea urged nurses to join the Nurses' National Total Abstinence League, and addresses were given by Dr. Sims Woodhead, Miss Orme (London Temperance Hospital), and others.—An excellent provision is included in the Public Health Acts Amendment Bill, just introduced into the House of Commons by Sir Alfred Hickman, a member for Wolverhampton. This is a clause empowering local authorities to employ nurses to attend on persons suffering from infectious diseases as occasion may arise.—The Empress of Russia has given a sum of 1,000,000 roubles for the building of residential quarters for women attending the Medical Institute at St. Petersburg.—A largely-attended public meeting was recently held at Omagh in aid of the District Nursing Society, at which an interesting address was given by Miss Dunn, superintendent of the Irish Branch of the Queen's Jubilee Institute for Nurses.—The sum of £1,000 has been given to Guy's Hospital by Mr. Charles Gassiot towards the cost of the new Nurses' Home about to be built.—The Crown Princess of Greece has ordered from Messrs. Leveson & Sons, 90 and 92, New Oxford Street, London, W.C., a number of Bath chairs and invalid carriages for the use of the Greek soldiers who have been wounded in the late war. These were forwarded this week to Athens by the s.s. *Craigmore*, from Liverpool.

### Pharmacy and Dispensing for Nurses.

By C. J. S. Thompson.

### XII.—SPECIAL EXCIPIENTS AND DIFFICULTIES IN PILL MAKING.—SILVERING AND COATING.

Chloride of ammonium is a difficult salt to work into a pill mass, but with the aid of a small quantity of soluble cream of tartar a good result can be obtained. Benzoic acid can be massed with Canada balsam, one to every four grains of the acid; or with glycerine, using one drop to five grains. Antipyrine with glycerine of tragacanth, or powdered gum and water. Nitrate of bismuth forms a good pill with soluble cream of tartar, or powdered gum tragacanth, and a few drops of water. Balsam of Peru with bread crumb or beeswax. Calomel may be made into a firm pill with the aid of confection of roses or manna and compound tragacanth powder. Calcined magnesia should not be used in rolling a mass containing calomel. In massing camphor, it should first be reduced to very fine powder with the aid of a few drops of spirit, then worked up with glycerine of tragacanth and powdered soap. Chloral hydrate must also be reduced to a very fine powder, when it may be massed with syrup, and powdered tragacanth, or Canada balsam, ½ gr. to 5 grs. of chloral, and made into pills. Ergotine, which is usually semi-liquid, should be very carefully evaporated over a water bath, and brought to a proper consistence by the addition of powdered gum acacia. Other thin extracts also require the addition of compound tragacanth powder or gum acacia to form a firm mass. Grey powder may be made into a satisfactory pill with confection of roses, care being exercised not to work it too hard, or the mercury will separate, and be found at the bottom of the mortar. Bromide and iodide of potassium, after being reduced to fine powder, form a good mass with liquorice powder, tragacanth, and a few drops of water, or with confection of roses. Permanganate of potassium decomposes when mixed with organic substances, therefore, after reducing to powder, it must be massed with kaolin or resin ointment. An excellent quinine pill can be made by adding one grain of tartaric acid to every ten grains of quinine and a drop of water.

Of the liquids that are likely to give the dispenser trouble, we will first consider creasote. Most dispensers have their favourite method of forming this body into a pill mass, but the success of the result depends largely on the manipulation. An excellent method of making a firm mass, is to well mix one part of creasote with five parts of liquorice powder and one part of powdered soap; another, with powdered liquorice and glycerine of tragacanth. A good pill may also be formed with calcium phosphate and hard soap as excipients. The student should practise all these methods until a good result is obtained.

Carbolic acid may be massed by incorporating two parts, with powdered althæa three parts, and glycerine a quarter. A firm pill may also be formed with liquorice and soap in the following proportions: Carbolic acid, one part; powdered soap, one part; liquorice powder, five parts. Balsam of copaiba should be mixed with calcined magnesia or slaked lime and allowed to stand for some hours, when it may be massed with the aid of a small quantity of bees-wax.

Essential oils require the addition of calcined magnesia and powdered soap, or calcium phosphate and soap, to form a workable mass.

Croton oil can be made into a firm pill with bread crumb, magnesia, and soap, or powdered liquorice and glycerine of tragacanth.

Tar should be massed with lycopodium, and oil of turpentine with calcined magnesia and white wax.

Phosphorus is a somewhat difficult substance to form into a satisfactory pill, and the student must be wary in handling it. In the first place a solvent is necessary, and in the second the solution must be incorporated with a suitable base. Bisulphide of carbon is one of the best solvents for phosphorus, but chloroform and theobroma oil answer the purpose well.

Phosphorus pills should be coated or varnished soon after being made, and kept in a dark place in well-closed amber-coloured bottles.

Of the various methods of finishing and coating pills used in pharmacy, silvering is the process most frequently employed by the dispenser.

The pills to be silvered should be kept free from powder and have a smooth surface. They should first be shaken up in a willow box or covered pot, with a drop or two of mucilage, until they have received a thin coating of the gum; then turn them into a clean pot in which a sheet or two of silver leaf has been placed, and rotate them rapidly for a few minutes. The superfluous silver may then be blown off and the pills placed into another clean pot, in which they should again be rapidly rotated for a few minutes. The process for varnishing pills is very simple, all that is necessary is to place them in a covered pot, with a small quantity of the varnish, and shake them for a few moments until they are evenly coated, then turn them out on a flat plate to dry. A good varnish can be made by dissolving one part of gum sandarach in one part of absolute alcohol.

To give pills a pearl coating is an operation that requires some dexterity, and is not by any means an easy one; but with practice good results may soon be obtained.

The following is a simple method: Moisten the pills by shaking them up in a pot with just sufficient of a mixture of mucilage of acacia, one drachm; syrup, one drachm; water, five drachms; spirit, one drachm; then turn them into a round bottomed covered pot about half filled with the following powder: Powdered French chalk, six drachms; and rice starch, two drachms. Rotate the covered pot rapidly and evenly until the pills have received a white coating, then transfer them to a clean pot without any powder, and rotate again until they are polished. Pills to be coated must be well rounded, hard, and free from powder.

Keratin coating is used for pills that are intended to pass through the stomach and into the small intestine before being dissolved. Keratin is prepared in alkaline and also in acid solution, the alkaline being employed for alkaline medicines, and the acid for those that are acid. Keratin is prepared by digesting the parings of horn with a liquid consisting of pepsin, one part; hydrochloric acid, one part; and water, 11 parts, so long as anything is removed. The residue is then dissolved in ammonia by prolonged maceration for several weeks, and the solution evaporated.

The alkaline solution is made by dissolving seven parts of this dried keratin in 100 parts of a mixture of equal volume of ammonia and alcohol, the acid solution of the same strength in acetic acid. A fatty excipient should be used for the pill mass, and no dusting powder, the pills being dipped in melted cocoa butter before coating. Coating is carried out by shaking the pills up in a covered pot with the solution, the process being repeated several times.

Pills may be coated with gelatine by placing each on the point of a fine needle, dipping them separately into a warm solution of gelatine, then placing the other end of the needle in a pincushion, and allowing them to dry and become hard. The gelatine solution for coating should be made as follows: Dissolve two parts of gelatine in eight parts of water over a water bath, and strain through muslin.

(To be continued.)

### Post-Graduate Clinics for Nurses.

By a Trained Nurse.

### XVI.—DR. WEIR MITCHELL ON "RESTFUL NURSES."

"There are some women," once said Dr. Weir Mitchell to me, "who could never become 'rest nurses' were they trained for half a lifetime. I know the right kind the moment they come into my office. And I have more than once engaged a woman without any training to look after some of my cases, just because she had the qualities for a rest nurse, and I have never had cause to regret taking this unusual step. It is easier in these special cases to dispense with skill and training—since so many of my patients need only to be kept in bed, surrounded by soothing influences, and systematically fed—than to dispense with the natural faculty for resting a sick person and giving just so much sympathy as is suitable to the condition and temperament of the patient. Some nurses have an atmosphere about them which sets one all on edge. In fact they make one feel as if one's nerves were being laid bare. Others, on the other hand, have an indefinable but very charming magnetic healing presence which—by itself—is the most valuable factor in the cure and care of the sick."

Coming from so great an authority on "nerves," these views are of special weight and interest. And, while no doubt it is true that Weir Mitchell cases need a specially high development in the nurse of soothing, restful qualities, it must be conceded that such gifts are of much value in the nursing of all classes of illness. Most medical cases—especially to-day, when "nerves" are more sensitive and more easily thrown off their balance owing to the complex and trying conditions of modern high-pressure life—are complicated to some degree by nervous or emotional disturbances. So that every nurse needs to be to some extent a "rest nurse." But, unfortunately, these restful qualities are just those which the modern nurse—in common with all modern types of woman—is in danger of losing. For does not the nurse follow her athletic sister on to the bicycle track and on to the golf links and leave there—as women who indulge in great muscular feats must ever do—some of the tenderness and sweetness which the up-to-date woman is bartering away—though it be the real birthright of woman—for the poor and empty triumph of a record run on a cycle or a crack shot with a golf-club?

Some years since Dr. Weir Mitchell was never tired of praising the quiet, soothing ways of the English woman, and he had no hesitation in appraising the British nurse as vastly superior to her American sister. "American women," he was wont to say, "are bundles of sensitive nerves, and are consequently no more fitted for nursing than they are for motherhood." He avers that only one American woman in ten is fitted by physical and nerve qualifications to be a mother—and for the same reason he holds that a much smaller proportion are suited to sick-nursing. The English woman of to-day is more and more approaching the American type, so far as nerve irritability and the absence of soothing qualities are concerned, and it will be a sorry day for the sick if nurses as a class desert the ways of womanliness and become mere muscular machines. Coming now to the consideration of restfulness in its practical relation to sickness, I would like to dwell on one or two points wherein the nurse may benefit her patient by learning to appreciate the times and seasons when "restative"—if one may coin the word—measures may be applied, or when stimulation is suitable. One cannot help noticing, more especially in the nursing of sick children, how much harm can be done by a constant aim towards "keeping them amused." Now it is a great strain to be kept bright, cheerful, and amused when we are quite well. But when we are ill, such entertainment is much more trying, for Nature is then doing her best to direct all the physical forces of the body towards healing that part which is diseased. And it seems to me there can be little doubt that, in keeping up so continual an effort to "brighten" sick people, much harm is and may be unconsciously effected. In the nursing of the sick—be they at home or in the hospital—there should be certain seasons set apart for silence and rest, so that the patient will automatically and unconsciously acquire the habit of repose at these periods. And this system of regular intervals of rest should apply to the sick irrespective of sex, age, or the nature of the illness.

I remember one delightful ward in my training school wherein the "Sister" used to insist on a "silent hour" from half-past two to half-past three daily It was the only ward in the hospital which enforced this golden rule, alike beneficial to the nurses fortunate enough to be drafted there and the patients therein, who flourished and blossomed forth into amazing convalescence under the silence *régime* and the good nursing which accompanied it. For the "Sister" was a born nurse, and she had the real sympathy and womanliness which caused her to appreciate the balm and healing of that one quiet hour in the day. It was a male surgical ward, and the patients on first admission used to chafe somewhat at "Sister's" dictum—against which there was no appeal—of "no conversation or newspaper reading during the hour." But gradually each patient came to appreciate this delicious lull in the busy hum of the ward. And "Sister" and her staff used to watch with satisfaction how patient after patient fell gradually under the soporific quiet of the time and went off into good, comfortable sleeps which frequently lasted long after the prescribed limit. And a rare good humour prevailed throughout such as I have never seen equalled in any other ward. The senior surgeon of the hospital was never tired of saying, "The patients here, Sister, get on as if by magic. What spells of witchcraft do you weave to heal them so quickly?" And though I was then but the newest of new probationers, I used to contrast the other wards of the hospital, with their restless, noisy afternoons, with the delightful restfulness which characterised ours, and felt so sorry for the really sick who could get no mid-day sleep or rest owing to the incessant conversations of their convalescent neighbours and other talkative patients. And I knew, with the natural instinct of a nurse, that the poor weary women in many wards would have been thankful if the children could have been stopped from play and chatter for just one little hour out of the long twenty-four. And, later on, when appointed as a "Sister" in a London hospital for children, I attempted to put into force the valuable lesson of a restful hour which the finest "Sister" with whom I had ever worked had imbued me. But traditions were too strong, and it had so long been the custom of this, as of all other children's hospitals, for the musical boxes and every form of stimulating amusement to be produced immediately the ward was "cleared" after the children's dinner, and everyone was so convinced that rattles and steam engines, whistles and noisy "fun" was the best for these little ones, many of whom had been awake since five a.m., that the attempt on my part to introduce a rest-hour was a complete failure. And visitors to the wards used to express their admiration and say it was "so sweet and charming to see the little ones having such a happy time." But in my heart of hearts I used to know it was bad nursing. And I longed to surround the little things with the quiet they so badly needed, and soothe their fractious, restless little minds and bodies.

### A Warning from the Cape.

THE influx of consumptive persons into the Cape Colony in search of health has increased in a marked manner during the last few years. The extremely dry, light air of the Karoos, of the Free State, and the Transvaal certainly has most beneficial effects in some cases; entire recovery has occasionally been chronicled, and more often patients have prolonged their lives by some years. There are, however, serious drawbacks attending this immigration of consumptives into the Colony. Doctors in the colony complain bitterly that consumption is thus introduced and spread, and that it has recently increased very much amongst the colonists themselves, and there is no doubt a great deal of truth in this.

Two very serious mistakes are made by those who send consumptive patients to the Colony. First, they send them too late. It is only in the very earliest stage of the disease that a cure can be hoped for, but this is a fact which people either shut their eyes to or really do not know. Some of these poor creatures hardly survive the voyage, and only land at Cape Town or Port Elizabeth to go straight into the hospital and die there. And the air of the sea coast, which is moist and enervating, is very bad for them; their only hope is in going a good way up country.

The second mistake that many persons make is in coming out here to work. They are poor, they cannot possibly afford to stay in the Colony unless they can earn their living, and they fancy that the air will immediately work such a wonderful change that they will be able to do work they cannot do in England. On arriving, however, they find that work such as they can do is not easily obtainable, competition being now severe out here, and that, even if they find employment, it may not be in a place where the climate is suitable for them, for there are many climatic variations in so large a country as South Africa.

A sickly youth, whose education has only fitted him for book-keeping, or something of that description, will gain little or nothing by emigrating. He will find the struggle for existence little, if at all, less severe than at home; while an indoor, sedentary life will prevent him from reaping due advantage from the climate; and, again, he will sorely miss the little comforts which are not obtainable out here except by fairly wealthy people, and miss these and the kindness and attentions of friends more and more bitterly as his disease gains on him, and he becomes gradually a helpless invalid.

It is nothing less than cruel to send out a boy or girl in an advanced stage of phthisis quite alone, with little money, to look for work six thousand miles from home and friends. Then, after a weary search for work, followed by weeks of illness, and dependent on the care of strangers; perhaps, as in one case of which I know, to die at last absolutely alone. This is no imaginary case, nor even a rare one; I am writing of what has been and is being done continually.

Those at home who are responsible for sending consumptive patients to the Cape Colony, should observe strictly these three points. Never to send them except in the very earliest stages of the disease. Never to send them alone. Never to send them out almost penniless, on the chance of getting employment. Consumptive persons and their friends are, as we know, always hopeful up to the very last; but they must remember that no climate can be an infallible cure, and that privations and discomforts will often more than counterbalance the benefits to be derived from the change of air.

### English Nurses in Greece.

THE following letter from one of the English nurses at present working out in Greece will interest our readers:—

"You will like to hear what our movements and experiences have been since we left England. We were travelling hard from the time we left Charing Cross till we reached Athens, a four days' journey, the route being via Folkestone, Boulogne, Paris, the Mont Cenis Pass, and Turin to Bologna, Ancona, and Brindisi. From here by boat to Corfu, and on to Patras, arriving a last at Athens on a Sunday evening. It was a beautiful journey all the way. Monday we spent in Athens seeing what we could while we waited for orders from head-quarters. We spent the morning at the Acropolis, going again to see it by moonlight. It is a beautiful sight, and you have a fine view of the city too. On Tuesday two of us were sent to Arta, or, rather, to Karavessera, near Arta. Retracing our steps to Patras, the Consul met us and took us to dine at his house, thence going straight on by boat and train to our destination. We met many of the volunteers and wounded returning, all looking in a pitiable plight. At Agrinion we lunched at the Mayor's house, and there met the special correspondent of the *Morning Post*. None of the Mayor's people could speak English, so we had to accept their hospitality in silence. The carriage in which we performed the rest of our journey was of a very primitive description, so we were tired out when we reached the hospital quite late. It was a cloudy night, but the fire-flies and glow-worms were lovely, and so was the scenery, the sight of the shepherds' fires and the sound of the guns giving a weird touch to the whole scene. We were soon on duty in the big hospital, but two of us were sent presently to take charge of a smaller one by the sea. Here we have sixteen beds. Our doctors are Greeks. The students work very well indeed, and are most kind to the patients. We have some bad cases, but most are doing well. The men seem to have a wonderful capacity for bearing pain, and many minor operations are done without any anæsthetic. They scarcely seem to feel, and pull themselves together in a marvellous way. The wounds do very well, they say because the men do not drink. Every antiseptic precaution possible is taken, and we have no erysipelas or tetanus here. We expect to be called back to Athens, where it is said the work is very heavy. Now there is an armistice for fifteen days, and we are trying to send all the cases we can to Athens, as there they can be far better attended to."

### Death in Our Ranks.

IT is with deep regret that we record the death of Nurse Charlotte (Mrs. Block), which took place at the London Temperance Hospital on May 23rd. She had contracted typhoid fever, it is supposed from one of the patients under her care, and from the first but faint hopes were entertained of her recovery, the attack being of a very virulent character. She passed peacefully away, adding another name to the list of devoted women who have laid down their lives for others. Whilst deploring the loss of a valuable life, the hospital must feel it a matter for thankfulness that this is the first death which has taken place amongst its nurses during the twenty-three and a half years of its existence.

### Minor Appointments.

KING'S NORTON NEW INFIRMARY.—Miss Mina Gibson Cowan has been selected to fill the post of Superintendent Nurse at this institution. Miss Cowan was trained at the Royal Infirmary, Glasgow, being subsequently appointed superintendent nurse at the Sunderland Union Infirmary, which post she leaves to take up her new work at King's Norton.

### Ladies' Waiting Rooms.

IN these days, when "everybody and his wife" sets forth on tours and travels with the expectation that such wanderings will be attended with all possible comforts and convenience, it is a curious circumstance that the ladies' waiting-rooms at the leading London railway stations should show so few signs of progression. Indeed, they would appear to be suffering from arrested development, as a round of the principal stations reveals a large percentage of their first-class ladies' rooms to be apartments of almost Stygian darkness, dusty, unventilated, and uninviting.

### CHARING CROSS.

The "distinguished foreigner" from the Continent gets a flattering first impression of London from the station situated so picturesquely in this historic spot. But the femininity belonging to such wayfarers of distinction on entering the first-class waiting-room provided for the purpose of enabling ladies to repair the ravages wrought in personal appearance by "a bad crossing" are wont to express themselves somewhat strongly on the inadequacies of the toilet accommodation provided at Charing Cross. In these dingy rooms, whose half-light makes the presentment of a face well-nigh impossible, the wear and tear of travel is so magnified and intensified by the general darkness and shortcomings of the toilet appurtenances, as to engender suicidal yearnings in the woman heart. And to these evils must be added a marked want of ventilation, and a general indifference to sanitation and her goodly laws. The construction of the station might cause difficulties in the way of admitting more daylight into the waiting-rooms, but a periodic "spring cleaning" and a supply of disinfectants is not too much to ask in the interests of the thousands of women passengers passing through this station, to whom long "waits" in such unhygienic rooms anticipate the headache and *malaise* which they expect as a legitimate consequence of crossing the Channel. Now that so many women qualify as sanitary inspectors it would be a simple matter for each railway company to appoint such an official, whose duty it would be to go the rounds of the "ladies' waiting-rooms" on their respective lines, and who would be held responsible for their general sanitation and cleanliness. An inspection of many of the innumerable country and wayside stations belonging to four principal railway lines leaving London, has revealed that in many of these every canon of wholesome and common-sense hygiene is broken in the dark interiors of their "ladies' waiting-rooms."

### WATERLOO STATION.

This important station was included in the tour of inspection undertaken a few days since by the writer of this article, anxious to gain information on a point so important to the travelling public. On that occasion a train stood ready to bear away the saloon freight of a steamer bound for South Africa, while an American liner "special" was steaming in the station. In consequence of these departures the first-class ladies' room was crowded, and a consensus of feminine opinion as to the adequacy of the waiting-room arrangements resulted in the "Noes" having an overwhelming majority. "The rooms are dark and dusty," said some. "We do not want to buy a wool mat or a gorgeous pincushion," said others, pointing to a bazaar-like array of such articles on the table. "We want to wash our hands—and there are at least seven candidates to each basin," wailed the dusty and travel worn. The small mirror accommodation was thronged by a crowd of ladies, tiptoeing over one another's shoulders, and elbowing for "a peep in the glass." A chorus of complaint was voiced by the throng longing in vain to adjust a hat-pin, a dishevelled fringe, or a disarranged veil. Such a scene once witnessed by even the sternest board of directors should be enough to effect a speedy reform in the methods of station waiting-rooms before the summer touring season sets in.

Paddington and Euston Stations possess fairly-equipped ladies' rooms, which would, however, be much improved by ventilation and disinfectants. At Euston the washing basins are of a size suitable only for the use of Liliputians, while hot water is a luxury compassed with great difficulty by the pleasant and obliging attendants, who boil the necessary supplies in small saucepans and kettles—their own private property.

To put these waiting-rooms in order is so simple and inexpensive a matter that no fractional diminution of railway dividends need take place. And well-lighted, wholesome, and sanitary toilet-rooms are not luxuries, but necessities. Certainly, generations of feminines would hold blessed that board of railway directors enterprising enough to put these at their disposal, and sufficiently public spirited to appoint a woman sanitary inspector to see that the standard of hygienic efficiency, once established, is kept up satisfactorily.

### Appointments.

### MATRONS.

THE NURSES' CO-OPERATION, 8, NEW CAVENDISH STREET, W.—Miss Amy Hughes has been appointed to succeed Miss Phillippa Hicks as Lady Superintendent of the Nurses' Co-operation. Miss Hughes received her training at St. Thomas's Hospital, and then joined the Metropolitan and National Nursing Association, subsequently being appointed superintendent of the Chelsea Home and then of the Central Home, Bloomsbury Square. Two years ago Miss Hughes resigned this post to take that of superintendent of nurses at Bolton Union Workhouse Hospital, where she has remained to the present date.

ENNISCORTHY DISTRICT LUNATIC ASYLUM.—Miss H. Ida Proschwitzky has been appointed to the position of Matron at this asylum. Miss Proschwitzky was trained at St. Marylebone Infirmary. For three and a half years she held the appointment of head nurse at the General Hospital, Darlington, and was then placed in charge of a floor at the East Dulwich Infirmary. She was also for two years sister at the Greek Hospital, Alexandria, and at the present time is superintendent of night nurses at the North-Western Hospital, Haverstock Hill.

ST. GEORGE'S HOSPITAL, S.W.—Miss Florence Smedley has been appointed to the post of Matron at this hospital, vacant through the retirement of Mrs. Coster. Miss Smedley received her training at St. Bartholomew's Hospital, where she held the position of ward sister from 1888 to 1893. From 1893 to 1894 Miss Smedley filled the post of matron at the Parkwood Convalescent Home, Swanley, since which date up to the present time she has been matron at the Hospital for Sick Children, Great Ormond Street.

PLYMOUTH BOROUGH HOSPITAL FOR INFECTIOUS DISEASES.—Miss Helen Butler-Browne has been appointed Matron at this hospital. Miss Butler-Browne received three years' training at the Birkenhead Borough Hospital, and has subsequently held appointments as matron of the Willesden Isolation Hospital, charge nurse at the M.A.B.'s South-Western Hospital, and night superintendent at Mill Road Infirmary, Liverpool.

INFECTIOUS DISEASES HOSPITAL, LEIGHTON BUZZARD.—Mrs. Sarah Franklin has been selected to fill the appointment of Nurse Matron at this hospital. Mrs. Franklin was trained at St. Thomas's Hospital, subsequently obtaining the appointment of head nurse at the Chertsey Isolation Hospital.

### The Book World for Women and Nurses.

[We invite Correspondence, Criticism, Enquiries, and Notes on Books likely to interest Women and Nurses. Address, Editor, The Hospital (Nurses' Book World), 28 & 29, Southampton Street, Strand, London, W.C.]

Antiseptic Principles for Nurses. By C. E. Richmond, F.R.C.S., Honorary Surgeon Ancoats Hospital, Manchester; Honorary Surgeon, Warrington Infirmary. (London: J. and A. Churchill. Pp. 47. Price 1s.)

The plan of this useful little book is directed towards supplementing the practical knowledge the nurse gains in the wards of the technique of antiseptic dressings by some instruction in the scientific principles which underly the theory of antiseptics. And such teaching is very necessary, for a nurse may apply sal-alembroth, and all the myriads of "dressings" which find favour for a day and are then superseded, without really grasping the inner meaning of a-sepsis. The average nurse hails from the average girl's school, where science is mostly doled out in homœopathic doses. And it is not surprising that a nurse so educated is apt to find for many months that the science teaching of her Training School lectures—simple though these may be—strikes her consciousness as more or less of a jargon and jumble of terms. On one occasion the writer recalls how a lecturer was laboriously explaining to a class of wondering probationers the nature of "germs," some colonies of which he demonstrated by lantern slides, offering at the same time the information that they were "broth cultures." Two Pros were afterwards heard declaring with horrified vigour that "they would never again touch beef-tea or soup since it contained such horrid live things." Dr. Richmond writes clearly and interestingly, but the book would be increased in value were it framed in more simple language. Many nurses read these little handbooks between times of active duty when mental activities are not at their clearest, and the pill of knowledge for them should be compounded in a light, assimilable form. The book will be popular, and if Dr. Richmond would simplify further editions, bearing in mind that the nurse has not had the preliminary training of a medical student, it is safe to predict a marked success for the book.

Lectures on Medicine to Nurses. By Herbert E. Cuff M.D. (London: J. and A. Churchill. 1896. Price 3s. 6d.)

This is a useful little work of rather more than 200 pages, giving a clear account of the main features of the more common forms of illness met with in private and hospital practice. Without entering into technical detail, the author affords in his lectures such information of the physiological, pathological, and therapeutic aspects of each case as should enable the nurse in charge to take a greater interest in her patient, and understand the phases present or to be expected during the course of the disease described.

Whitaker's Directory of Titled Persons, 1897.

We have tested in several points the efficiency and thoroughness with which this volume has been compiled, with the results one would expect from the source whence the directory emanates. As a trustworthy book of reference this is cleverly reduced to limits of reasonable proportions, being the same in size and appearance as the immortal "Whitaker Almanack," which it is designed to be a companion volume on the library table. The year of its first issue being coincident with the sixtieth anniversary of Her Majesty's accession, it was deemed fitting for supplying the full particulars which will be found under this division; full historical and descriptive accounts of the Royal Family are here, as of the Peerage, Baronetage, and Knightage. The Directory bears the stamp of accurate compiling, and we venture to prophecy will only need to be known to be regarded as a standard work, essential to every cultivated home. It is published at 12, Warwick Lane, Paternoster Row, E.C., at the modest charge of 2s. 6d.

### Everybody's Opinion.

[Correspondence on all subjects is invited, but we cannot in any way be responsible for the opinions expressed by our correspondents. No communication can be entertained if the name and address of the correspondent is not given, or unless one side of the paper only is written on.]

NURSES AND CYCLING.

"E. R. W." writes: In answer to the letters in your last issue with reference to nurses who bicycle, I should just like to say that six months ago I should have written even more strongly on the subject. My father brought me up with such a holy horror for masculine women that I almost felt it a religious duty to form a crusade against bicycling women—let alone bicycling nurses; so that when my committee and the doctor suggested my having one, I drew myself up to my full length and said I would sooner walk my feet off than be seen by my patients to be jumping up on two wheels. Time passed on, and I daily grew more obdurate on the subject, when suddenly one morning my own father presented me with one—"a beauty," as all connoisseurs called it. The committee laughed at me, and the two doctors offered to come and teach me, but still I held out; once a week I used to go on the sly and have a look at the thing, and come away shaking my head and saying, "I really cannot do it; what will my patients say?" Finally I made up my mind to go to the neighbouring town and secretly to learn how to "bike," thinking that some day in an emergency I might be glad to use it, and thus perhaps to save a life. Gradually I began to use it in the village, but I felt so uncomfortable and self-conscious at first that I always dismounted if I happened to see any one coming along the road. Nobody but myself knows what I suffered. But now—shall I acknowledge it?—I fly about everywhere on my wheels. I go for rides with members of the committee, the doctors, the clergy, and their wives I wear a navy blue uniform dress (the skirt is narrow and short) and a bonnet without a veil, and personally I do not see why a nurse should not be seen cycling any more than a clergyman or a doctor. We wear our uniforms for convenience and recognition, and not to pose as angels. I have met nurses who are splendid women and beautiful types of womanhood, but in no way have they posed as a higher order of humanity from their married friends, who though living in the world are being just as devoted and hard-working and self-sacrificing as wives, mothers, and kind, thoughtful mistresses to their servants, and are in their way leading as noble lives as any nurse I know. All we claim to be is that we are women with a purpose in life, and as long as we allow nothing to weaken that purpose, we are perfectly consistent in whatever we chose to do in keeping up our health, whether it is riding a bicycle or sitting on the top of an omnibus, and I feel certain that in another year the thing will become so common that nobody will even comment on it.

### Jubilee Arrangements for Nurses.

Victoria Commemoration Club, 29, Southampton Street, Strand, W.C.—Special arrangements have been made at this club for the benefit of nurses during Jubilee week. Meals will be provided at the following prices: On June 21st and 23rd—Lunch, 2s. 6d.; tea, 1s.; dinner, 3s. 6d. On June 22nd—Lunch, 5s.; tea, 1s.; dinner, 3s. 6d. Tickets may be had for each meal separately, or an inclusive ticket for the three meals, 8s. 6d. Early application must be made to the secretary for all tickets, as in no case can tickets be issued after the morning of June 18th. Inclusive tickets for the three days' meals may be obtained for one guinea per person. Members may invite their friends, who will be provided for at the same rate.

### For Reading to the Sick.

WHITSUNTIDE.

THE Comforter, which is the Holy Ghost, Whom the Father will send in My name.

—*St. John*, xiv. 26.

**Verses.**

Come, Holy Ghost, our souls inspire,
And lighten with Celestial fire;
Thou the Anointing Spirit art
Who dost Thy sevenfold gifts impart.
Thy blessed unction from above
Is comfort, life, and fire of love;
Enable with perpetual light
The dulness of our blinded sight.
Anoint and cheer our soiled face
With the abundance of Thy grace:
Keep far our foes, give peace at home;
Where Thou art Guide no ill can come.
Teach us to know the Father, Son,
And Thee, of Both, to be the One;
That through the ages all along
This may be our endless song,
Praise to thine eternal merit,
Father, Son, and Holy Spirit.—*Latin Hymn.*

O Saving Victim, opening wide
The gate of heaven to man below,
Our foes press in on every side;
Thine aid supply, Thy strength bestow.

To Thy great Name be endless praise,
Immortal Godhead, One in Three!
O grant us endless length of days,
In our true native land, with Thee.

The very name of Comforter, or Advocate, how much is contained in it, how much that outweighs all the cares, all the opposition, all the sufferings of the world; to have One with us Who is God. Who is sent specially for this purpose to be the strength and refuge of those that believe in Christ. Nor is there less in that other Name, the Spirit of Truth. How do falsehood and disguise, how do all the deceits of the world, and all the arts of the father of lies, and the unrealities, vanities, and fleeting shadows of the world, flee away at the very name, the Spirit of Truth!—*Isaac Williams.*

I will pray the Father, and He shall give you another Comforter, that He may abide with you for ever; even the Spirit of Truth.—*St. John* xiv. 16, 17.

I will not leave you comfortless; I will come to you.—*St. John* xiv. 18.

Christ's prayer was, "Father give them the Holy Spirit to teach, sanctify, and comfort them." His Father should send, He said; and His Father did send, and the Holy Ghost came to-day. And came in that sort whereof they had most need, a "comforter." If we ask, Why under that term? to show the peculiar end to which He came. If they had been perplexed, "the Spirit of Truth." If in pollution of sin, "the sanctifying Spirit." But to-day they were as orphans, cast down and comfortless, their hearts full of heaviness. It was comfort they wanted: a comforter to them was worth all.—*Bishop Andrewes.*

### Wants and Workers.

[THE attention of correspondents is directed to the fact that "Helps in Sickness and to Health" (Scientific Press, 28 & 29, Southampton Street, Strand, London, W.C.) will enable them promptly to find the most suitable accommodation for difficult or special cases.]

DISTRICT NURSE, All Saints, Whetstone, wishes to obtain votes for the Royal Female Orphan Asylum, Beddington, for an urgent case, which will be superannuated unless successful at the June election, and will be very grateful for help.

### Notes and Queries.

**The contents of the Editor's Letter-box have now reached such unwieldy proportions that it has become necessary to establish a hard and fast rule regarding Answers to Correspondents. In future, all questions requiring replies will continue to be answered in this column without any fee. If an answer is required by letter, a fee of half-a-crown must be enclosed with the note containing the enquiry. We are always pleased to help our numerous correspondents to the fullest extent, and we can trust them to sympathise in the overwhelming amount of writing which makes the new rules a necessity. Every communication must be accompanied by the writer's name and address, otherwise it will receive no attention.**

*Paraplegia.*

(276) Can you tell me if there are any hospitals or homes in or near London where a young man with paraplegia could be taken in? He is in receipt of a small pension.—*Violet.*

A certain number of cases are admitted at the National Hospital for Paralysis in Queen Square, W.C., and at the National Hospital for Diseases of the Heart and Paralysis, 32, Soho Square, W., on payment of a weekly sum. At the Hospital for Epilepsy and Paralysis, Portland Terrace, Regent's Park, patients may pay according to means. You had better apply to these institutions for full particulars as to admission.

*Sanitary Inspectorships.*

(277) Can anyone kindly tell me whether ordinary elementary education is sufficient for a lady sanitary inspector, and advise me as to books to study?—*A. P.*

Write to Miss Lamport, 52, St. John's Wood Road, who trains ladies for sanitary work, or apply at Bedford College, Baker Street.

(278) Please tell me the address of the secretary of the Army Nursing Reserve, or tell me to whom I should apply to have my name enrolled as a member?—*E. A. C.*

You must apply at the War Office for all particulars, regulations, &c.

*A Question of Maintenance.*

(279) Can any readers of THE HOSPITAL tell me their experience as to what is a fair and comfortable allowance per nurse per week for boarding in a district home? The home in question contains four nurses and superintendent. The price of food is much the same as in large towns; fish and vegetables dear. Is 10s. for board only too much? No beer or stimulants.—*District Superintendent.*

Perhaps some of our readers will give "District Superintendent" the benefit of their views on this matter.

*District Training.*

(280) Will you kindly tell me if there is any place where training can be received in district nursing other than through the Queen's Jubilee Institute for Nurses? I am already trained in medical and surgical nursing.—*A. H.*

Write to Sister Katherine Twining, District Nurses' Home, Howard's Road, Plaistow, E., or to Miss Grey, Central Home, Metropolitan Nursing Association, 17, Bloomsbury Square, W.C.

*A Case for Advice.*

(281) I am a monthly nurse and was engaged some time since for a case at the end of April or beginning of May. It has not come off yet (May 23rd), and while I have been waiting I have had to give up another case for this month. I have asked to be paid from the 2nd of this month, but have been refused. Can I claim the full amount, or how much? Or could I take legal steps? Please advise me.—*Nurse.*

We believe that if you have full proof of your engagement you can claim the full fee from the date of engagement. It is, however, better to make a compromise and accept half or two-thirds of the fee rather than go to law on the matter.

*Annual Leave.*

(282) Please advise me in the following circumstances: A month's holiday annually is promised to a district nurse, but bad and heavy cases prevent its being taken. The committee promise it the beginning of the second year, but at the end of the first year the nurse resigns. Can she claim any part of it during the month of notice? Also can a committee compel her to allow her house to be made into a meeting-place weekly for visiting committee without her consent?—*Enquirer.*

In the case of a Queen's nurse such a state of affairs could not happen, it being the invariable custom for a nurse's holiday to be arranged for during the summer months. If the district cannot be left a temporary nurse is engaged for the month by the committee so that in any case her proper holiday cannot be interfered with. Apparently in your case the committee have broken faith with you in not arranging for the promised month, and if you did not leave until you had completed a year's work you could, we should think, certainly claim compensation, *i.e*, salary for one month. With regard to your second question it is difficult to reply, not being in possession of all the circumstances. In the branches of the Queen's Jubilee Institute the committees meet once a month, and usually at the rooms of the nurse, who as a rule is proud to have the meetings there, because she finds it helps to interest the committee in her work. The arrangement should have been clearly settled one way or the other at the beginning of your engagement. Instances like this show very clearly the great advantage it is to district nurses to belong to a body like the Queen's Jubilee Institute, which by clear and definite regulations prevents the possibility of friction between a nurse and her committee.

